# A Case of Disseminated Melioidosis With Cerebritis

**DOI:** 10.7759/cureus.40182

**Published:** 2023-06-09

**Authors:** Vaibhav Bhat, Siddharth Gosavi, Gokul Krishnan, Raviraja V Acharya

**Affiliations:** 1 Internal Medicine, Kasturba Medical College of Manipal, Manipal, IND

**Keywords:** india, s: burkholderia pseudomallei, dual antibiotic, cerebritis, melioidosis

## Abstract

Melioidosis is caused by *Burkholderia pseudomallei, *a Gram-negative, facultative intracellular bacterium. Because melioidosis can mimic many diseases, it requires more advanced laboratory facilities with the necessary expertise and can become an underdiagnosed yet serious infection with high mortality and morbidity*. *Our patient is a middle-aged male with new-onset uncontrolled type 2 diabetes mellitus who presented with high-grade fever, productive cough and altered mental status. CT thorax showed diffuse middle and lower zone consolidation while MRI brain noted meningitis with cerebritis. Blood culture grew* Burkholderia pseudomallei*. The patient was started on meropenem for melioidosis, however, no adequate improvement was seen. In view of this inadequate response, parenteral cotrimoxazole was added. Significant improvement was noted and cotrimoxazole was continued for six months.

## Introduction

Melioidosis is caused by *Burkholderia pseudomallei *(*B. pseudomallei*), a Gram-negative, facultative intracellular bacterium. It is predominantly endemic to South Asia including the Indian subcontinent, Southeast Asia, and Northern Australia with sporadic non-travel-associated cases being reported from the US and African countries [1]. Neurological involvement is rare, accounting for 4% of cases [2]. We report one such case of neuromelioidosis in an adult male with newly diagnosed type 2 diabetes mellitus. He was managed successfully with antibiotic therapy comprising meropenem and cotrimoxazole.

## Case presentation

A 43-year-old male farmer newly detected with uncontrolled type 2 diabetes mellitus presented with complaints of high-grade fever for 10 days which was associated with chills and rigours. It was intermittent, temporarily relieved by over-the-counter medications like acetaminophen. The patient also complained of progressive cough with mucopurulent expectoration for seven days. He started to experience episodes of severe headaches along with blurring of vision for seven days. His brother noticed irritability, irrelevant talk and drowsiness five days prior to his presentation to the hospital. He soon developed worsening dyspnea over the last two days prior to his visit to our centre. He presented to the primary healthcare centre with the above-mentioned complaints and was referred to our tertiary hospital on the same day in view of poor sensorium, hypoxia and hypotension.

On arrival, the patient had a Glasgow Coma Scale of E3 V2 M5, with an intact airway [3]. Vital parameters were deranged with hypotension (blood pressure 80/50 mmHg and tachycardia (heart rate 120/min). Oxygen saturation of 90% was noted on room air, with a respiratory rate of 28 cycles/min. On systemic examination, respiratory examination showed left-sided decreased chest movements with central trachea, normal palpatory and percussion findings; auscultation revealed left-sided tubular bronchial breath sounds with coarse crepitations in infra-scapular and infra-axillary areas. Neurological examination showed disorientation and irritability with no focal neurological deficits or signs of meningeal irritation. As mentioned above, this patient with uncontrolled diabetes presenting with features of pneumonia and meningoencephalitis prompted us to take disseminated melioidosis as a differential diagnosis.

Investigations

Initial laboratory investigations revealed bicytopenia, acute kidney injury, transaminitis and newly detected type 2 diabetes mellitus (Tables 1-2). Arterial blood gas analysis was suggestive of hypoxia with metabolic acidosis and respiratory alkalosis. Urine ketones were negative. Blood culture performed by automated BacT/ALERT method taken from different sites grew *B. pseudomallei* with sensitivity report as mentioned (Table 3). Three sets of blood cultures performed on Days 1, 2 and 5 as per Clinical and Laboratory Standards Institute (CLSI) standards were positive. Sputum acid-fast bacilli and GeneXpert tests carried out to look for tuberculosis were negative.

**Table 1:** Haemogram at admission and discharge

Investigations	At Admission	At Discharge
Haemoglobin	12.9 g/dL	11.1 g/dL
Total leukocyte count	3100 /microlitre	6200 /microlitre
Platelet counts	35,000 /microlitre	2,07,000 /microlitre

**Table 2:** Laboratory parameters at admission and discharge CRP: C-reactive protein; INR: international normalized ratio; PT: prothrombin time; APTT: activated partial thromboplastin time

­­­­­­Investigations	At Admission	Day 7	Day 14	At Discharge(day 18)
HbA1c	10%	-	-	-
Blood glucose	259 mg/dL	-	-	
Urea (mg/dL)	93	22	21	21
Creatinine (mg/dL)	2.56	0.78	0.77	0.77
Sodium (mmol/L)	139	127	130	130
Potassium (mmol/L)	3.6	3.7	4.7	4.7
Total bilirubin (mg/dL)	8.57	7.74	2.25	1.65
Direct bilirubin (mg/dL)	5.97	7.17	1.89	1.39
Aspartate transaminase (IU/L)	82	48	28	42
Alanine transaminase (IU/L)	37	39	44	49
Alkaline phosphatase (IU/L)	818	648	962	852
Albumin (g/dL)	2.82	-	-	3
Globulin (g/dL)	2.8	-	-	4.3
CRP	291	140	-	105
INR	1.07	-	-	-
PT (seconds)	12	-	-	-
APTT (seconds)	27.2	-	-	-
Serum bicarbonate (mEq/L)	20	-	21	-
pO2 (mmHg)	66	-	97	-
pCO2 (mmHg)	33	-	29	-

**Table 3:** Blood culture and sensitivity report

Bottle A	Burkholderia pseudomallei
Bottle B	Burkholderia pseudomallei
Antibiotic sensitivity	
Amoxicillin-Clavulanic Acid	Sensitive
Ceftazidime	Sensitive
Trimethoprim sulfamethoxazole	Sensitive
Gentamicin	Resistant
Doxycycline	Sensitive
Colistin	Resistant
Meropenem	Sensitive

**Figure 1:**
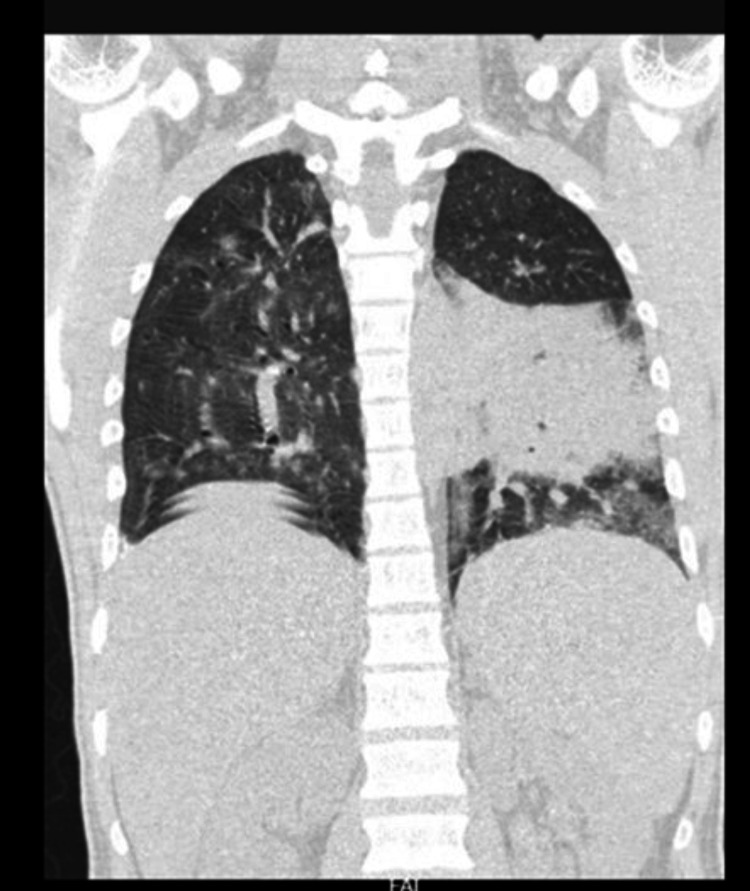
CT thorax-Coronal section showing a large near-homogenous hyperattenuating area in the left lower lobe with surrounding patchy ground-glass attenuation suggestive of consolidation

**Figure 2:**
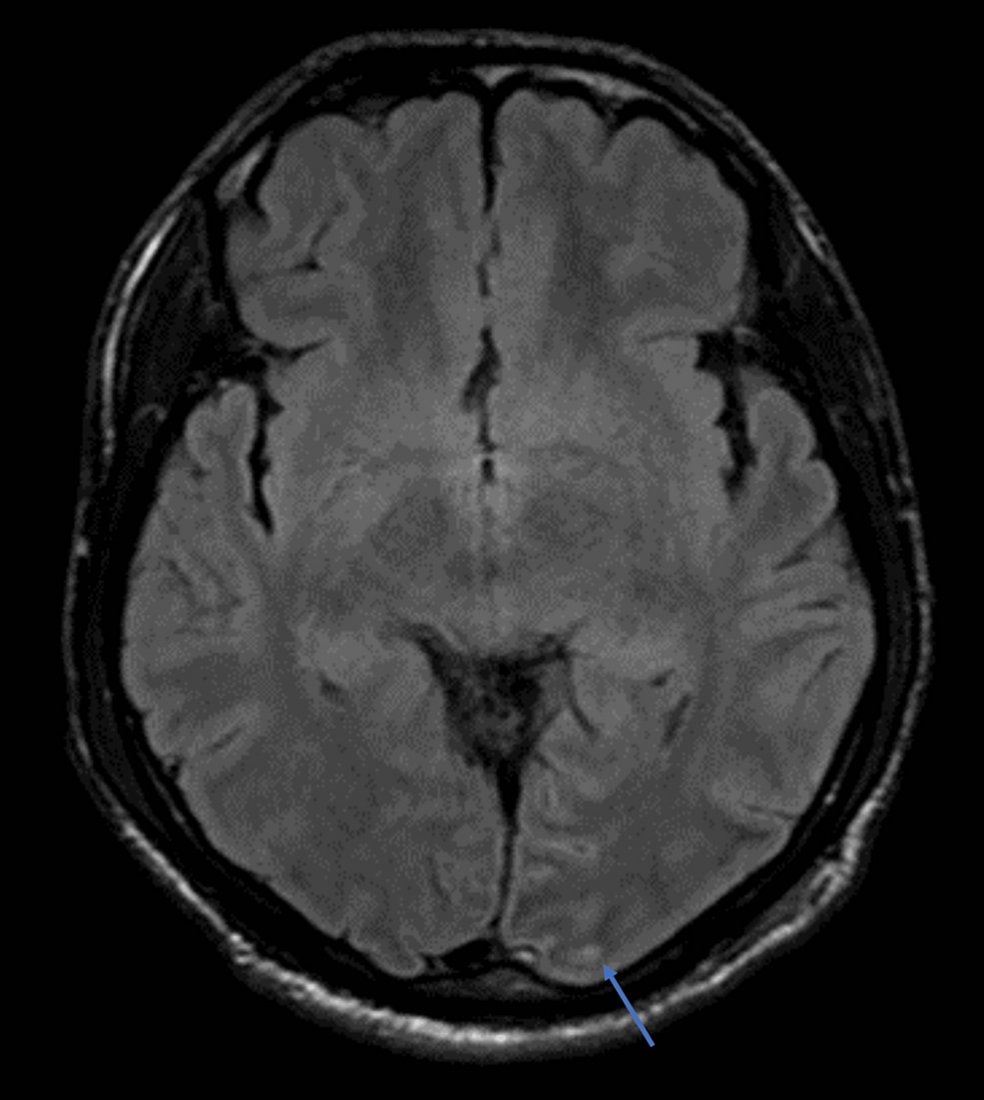
MRI brain showing FLAIR hyperintensities along the sulci of the left parieto-occipital region with mild post-contrast enhancement (see arrow)

Ultrasound abdomen showed hepatosplenomegaly. High-resolution CT thorax showed features of consolidation of the left lower lobe (Figure 1). MRI brain with contrast performed in view of persisting poor sensorium was suggestive of cerebritis with meningeal enhancement (Figure 2). Lumbar puncture and CSF analysis were deferred as the patient had severe thrombocytopenia.

Differential diagnosis

Community-acquired pneumonia with sepsis leading to shock was considered as a differential in view of high-grade fever and cough with purulent expectoration. Altered mental status was thought to be secondary to hypotension and sepsis. Disseminated tuberculosis affecting predominantly lungs and brain was thought of as a provisional diagnosis considering endemicity along with fever, cough and altered mental status. Disseminated melioidosis was also considered as he had uncontrolled diabetes mellitus and features of sepsis.

Treatment

The patient was admitted to the intensive care unit and started on non-invasive ventilation. Supportive management was provided in the form of parenteral fluids and inotropic support for septic shock. An initial laboratory workup with blood and sputum cultures was sent and the patient was started on intravenous piperacillin-tazobactam (as per renal dosing) and azithromycin. It was escalated to intravenous meropenem as per blood culture reports within 24 hours of admission which grew *B. pseudomallei*. As no improvement of shock or oxygenation was seen, intravenous cotrimoxazole was added on day four of the hospital stay. Serial blood cultures were done till culture negativity was noted. The first sterile blood culture was reported on Day 8. A repeat blood culture was sent at discharge, which was sterile. Renal impairment was managed conservatively with hydration and management of underlying infection. Improvement in renal function was seen with this approach. Glycaemic control was achieved with subcutaneous short-acting insulin. The patient was also started on chest physiotherapy and limb physiotherapy for improvement of respiratory efforts.

Outcome and follow up

The patient improved clinically and was discharged after 18 days of intravenous meropenem and 14 days of intravenous cotrimoxazole, followed by oral cotrimoxazole for six months. The patient was followed up with complete blood counts, renal function tests with potassium and liver function tests after two weeks and one-month post-discharge. At two weeks and one month, the patient had normal biochemical parameters and resumed his occupation but with lesser physical exertion.

## Discussion

*B. pseudomallei* is the Gram-negative bacterium responsible for melioidosis. It is considered epizootic in nature in the southern states bordering the Western and Eastern Ghats of India but the full extent of spread in the rest of the country is not yet known [4]. Transmission can occur by percutaneous inoculation, aspiration, inhalation and rarely ingestion. The predominant mode of transmission is believed to be the percutaneous route due to exposure to contaminated soil (wet, especially during monsoon) and contaminated water [5]. However, during climate catastrophes the main mode is inhalation. Documented evidence of an increase in melioidosis with pneumonia was observed post such events [6]. Ingestion is an important route of transmission in endemic areas due to contaminated water, seen commonly in Southeast Asian countries compared to the Australian continent [5]. Person-to-person mode of transmission is rare and transmission of melioidosis via sexual contact remains unproven [7,8].

It was seen that severe melioidosis or rapidly progressive sepsis was reported in patients with the following notable comorbidities such as uncontrolled diabetes mellitus, chronic alcohol abuse, chronic kidney disease and chronic lung disease [9,10]. A study from Northern Australia showed that the absence of risk factors was associated with higher chances of survival [11]. Melioidosis very frequently presents with lower respiratory tract infections in the form of pneumonia [12]. Some of the other sites seen were skin, soft tissues and the genitourinary system. The majority of the patients progress to septic shock with mortality ranging from 10 to 40% [13].

There are few documented cases from developing countries like India due to diagnostic and infrastructural challenges. We present to you one such case of disseminated melioidosis presenting as meningitis with cerebritis and pneumonia. Pneumonia is one of the most common presentations, often manifesting as acute community-acquired pneumonia. It usually progresses to multi-organ involvement. Common chest radiographic findings are diffuse or patchy non-homogenous shadows involving a single lobe or as a multi-lobar disease. Other findings usually seen are cavitation and abscesses [6]. Our patient had lung involvement characterised by lower lobe involvement requiring oxygen support initially. In India, these findings pose a great challenge as it is a close differential to a very commonly seen disease all over the country, tuberculosis. 

Skin infection is another relatively common presentation of this disease. They present as ulcers, superficial abscesses, pustules/ furuncles and macules. Cutaneous melioidosis is usually suspected when usual skin lesions are unresponsive to commonly used antibiotics. Rarely do they present as cellulitis [14]. Other important modes of presentation are osteomyelitis and septic arthritis. It is usually a part of disseminated melioidosis as a secondary manifestation due to another primary site, usually pneumonia, or rarely as a primary manifestation [8].

Melioidosis occasionally involves the central nervous system. Nervous system involvement can be in the form of meningitis, meningoencephalitis, cerebritis, cerebral abscess and myelitis. Cases of epidural abscesses have also been noted. In Southeast Asia, cerebral abscess due to disseminated melioidosis is the most common neurological manifestation accounting for approximately 4% of melioidosis cases while in northern Australia, encephalomyelitis is the predominant manifestation. Presenting symptoms can be in the form of seizures, and focal neurological deficits like limb weakness or delirium [2,15]. Cranial nerve palsies are also known to occur [16]. The route of spread can be hematogenous, with brainstem invasion through the nasal route and cranial nerves. Occasionally, the nervous system can be involved by spreading through the percutaneous route [9,17]. Our patient was initially treated with meropenem following blood culture reports which showed growth of *B. pseudomallei*. However, satisfactory improvement was not observed clinically and as per laboratory parameters. The patient was started on intravenous cotrimoxazole as soon as this was noticed. Following the addition of cotrimoxazole, a better response was noted with recovery from shock and respiratory failure. The patient was eventually weaned off ventilation and ionotropic supports. In cases of neuromelioidosis, we can see that early initiation of dual antibiotics gives excellent response instead of waiting for CSF analysis or MRI studies. Cotrimoxazole is indicated during the intensive phase in cases of non-pulmonary foci of infection. The rationale behind the addition of cotrimoxazole is to improve tissue penetration and reduce the development of resistance [18].

We would like to emphasize on early initiation of carbapenem with cotrimoxazole in severe cases of melioidosis (especially neuromelioidosis). *B. pseudomallei* is known to cause bone marrow suppression, which was noted in this patient where he presented with bicytopenia - anaemia with severe thrombocytopenia. In such scenarios, correction of thrombocytopenia for CSF analysis or MRI under sedation for definitive CNS diagnosis might delay appropriate therapy, thereby causing the progression of the disease which is known to have a high mortality and morbidity.

## Conclusions

There should be a high index of suspicion for melioidosis while treating patients with sepsis, especially for neuromelioidosis when comorbidities, as in this case, are present. Early CNS imaging is advisable in patients with suspected neuromelioidosis. We would also like to stress the importance of dual antibiotic therapy in neuromelioidosis.
